# Standardized naming convention and classification system for critical loads of nitrogen and sulfur deposition

**DOI:** 10.1002/ecs2.4473

**Published:** 2023-06

**Authors:** Jennifer Phelan, Michael D. Bell, Jason A. Lynch, Linda H. Geiser

**Affiliations:** 1 RTI International, Research Triangle Park, North Carolina, USA; 2 National Park Service – Air Resources Division, Lakewood, Colorado, USA; 3 US Environmental Protection Agency – Office of Air and Radiation, Office of Atmospheric Protection, Washington, DC, USA; 4 US Department of Agriculture – Forest Service, Air Resource Management, Washington, DC, USA

**Keywords:** classification, critical load, deposition, name, nitrogen, sulfur

## Abstract

Critical loads (CLs) of atmospheric deposition have been used for multiple decades to assess the impacts of air pollutants on terrestrial and aquatic ecosystems. However, these CLs have been developed by different researchers, at different times, using different methods, and are named in different ways with varying levels of information and levels of specificity. In this study, we identified the elements that describe CLs and used them to develop and test a standardized CL naming convention and a complementary CL classification system applicable to all CLs. The CL naming convention consists of: the form of deposition; the biological receptor, response, and threshold; and chemical criterion and threshold, thereby clearly communicating what the CL is and what it protects. The CL classification system contains all the elements that define and describe a CL, but the CL classes are not fixed and are therefore, defined by the user. The application of the CL naming convention and classification system to an existing US CL database and national forest was successful, demonstrating not only the utility of the naming convention and classification system, but how both can also be used to guide future CL science.

## INTRODUCTION

Human activity, predominantly through agriculture and fossil fuel combustion, has dramatically altered the natural distribution and cycling of nitrogen (N) and sulfur (S), and has led to significant increases in the emissions and air pollution deposition of the two elements (Doney et al., 2007; Galloway et al., 2004, 2008; Rodhe, 1999; Vitousek et al., 1997). Although N and S are essential elements for the growth and productivity of biota, elevated levels of N and S deposition, either individually or interactively, can negatively impact terrestrial and aquatic ecosystems.

Both N and S act as acidifying agents, and in terrestrial systems, deposition of N and/or S can cause base cation leaching from soils, decreases in soil pH, mobilization of phytotoxic soil aluminum (Al), and nutrient imbalances (Bobbink et al., 2010; Driscoll et al., 2003; Horswill et al., 2008; Lawrence et al., 2015; Pardo et al., 2011; Warby et al., 2009). Changes in soil chemistry ultimately harm sensitive terrestrial biota and cause alterations in plant, lichen, and fungal diversity (Chen et al., 2018; Geiser et al., 2019, 2021; Pan et al., 2020; Root et al., 2015; Simkin et al., 2016) and impair tree growth, survival, and recruitment (Dietze & Moorcroft, 2011; Duarte et al., 2013; Horn et al., 2018; Sullivan et al., 2013). In addition, N and S deposition can result in direct acidification and increased leaching of acidifying compounds into aquatic systems, thereby altering the chemistry of surface waters and adversely impacting the diversity of fish species and aquatic biota (Baldigo et al., 2018; Driscoll et al., 2001; Nierzwicki-Bauer et al., 2010).

Nitrogen also frequently limits the productivity of many terrestrial and aquatic ecosystems (Aber et al., 1998; Aerts & Chapin, 2000; Howarth, 2008; Vitousek & Howarth, 1991), and thus, elevated N deposition often stimulates growth (Du & de Vries, 2018; Vitousek & Howarth, 1991). Higher inputs of N, however, can also lead to eutrophication and decreases in plant diversity (Simkin et al., 2016) and shifts in forest composition due to changes in growth, survival, and competitive advantages (Bobbink et al., 2010; Emmett, 2007; Horn et al., 2018; Thomas et al., 2010). Furthermore, once a site becomes N saturated, and N availability is greater than the combined demands of plants and microbes, excess N is leached from soils into surface waters, harming downstream aquatic ecosystems and aquatic flora and fauna (Aber et al., 1998; Driscoll et al., 2003; Emmett, 2007).

Scientists have therefore developed the concept of a “critical load” (CL), which is defined as “the quantitative estimate of an exposure to one or more pollutants below which significant harmful effects on specified sensitive elements of the environment are not expected to occur according to present knowledge” (Nilsson & Grennfelt, 1988). CLs provide scientists, managers, and policy makers a means to gauge the current and future protection of natural resources against air pollution and the resulting deposition effects. Thus, CLs of N and/or S deposition have been used extensively in Europe to support the protocols under the Convention on the Long-range Transboundary Air Pollution (LRTAP) (Hettelingh et al., 2015) and more recently by federal land managers in the United States in the development of management plans and setting goals for resource protection and restoration in national parks, national forests, and other federal lands (McMurray et al., 2021; Porter et al., 2005). The U.S. Environmental Protection Agency (EPA) is also using CLs in their evaluation of the National Ambient Air Quality Standards (NAAQS) for N and S oxides and particulate matter (PM) (https://cfpub.epa.gov/si/si_public_record_report.cfm?Lab=OAQPS&dirEntryID=338055).

CLs can be determined in different ways (de Vries et al., 2015). Many are estimated through empirical dose–response relationships or deposition gradient analyses that directly relate deposition of N and/or S additions to measured biotic and abiotic responses in the field. Such empirical CLs are based on an amount or threshold of change to a biological receptor (e.g., herbaceous species richness) or chemical indicator (e.g., soil nitrate leaching) and the corresponding level of deposition. The gradient of deposition can be from N additions, a transect away from a source location, or datapoints collected across a range of deposition values. Other CLs are estimated with mass balance and process-based biogeochemical models that account for differences in system inputs and outputs and protect against adverse changes in soil solution or surface water chemical conditions that negatively impact biota. These CLs are based on relationships between soil/water chemistry and the condition of biota within the terrestrial or aquatic system to protect either a broad spectrum of species within taxa groups (e.g., all trees) or specific species with known sensitivities under certain chemical conditions (e.g., brook trout).

The various methods to estimate CLs have, therefore, resulted in the naming of CLs in different ways. Acidification CLs based on steady-state mass balance models are commonly specified as forest soil, terrestrial, surface water, or aquatic acidification CLs. Empirical CLs have titles such as “CL of N for nitrate (NO_3_^−^) leaching” or “CL for mycorrhizal fungi.” CLs are also given more generic labels like “CL of acidity,” “CL of nutrient N,” “CL of eutrophication,” “CLs of biodiversity,” and “empirical CL for N” to group them by estimation method or apparent mechanism of impact (CLRTAP, 2017; Hettelingh et al., 2015; Lynch et al., 2022; Pardo et al., 2011). Although such labels may be easy and make sense to the scientists who have developed them, they are not consistent and may not be as clear to nonscientists, natural resource managers, or policy makers who use CLs to support management and policy decisions. Essentially, many of these labels or CL names are simplified and do not include important information. For example, it is not apparent from its name what biological or ecological protections are provided by a “forest soil acidification CL” or “CL of N for NO_3_^−^ leaching” or what biological response occurs when the CL is exceeded. Similarly, although a CL of N for mycorrhizal fungi indicates what is being protected, the response of the fungi to elevated N is not obvious. It may be associated with a reduction in diversity or an increase in fungal tissue N concentration, two response metrics that have potentially different implications for the fungal and forest community. Although the lack of a name that clearly indicates what the CL is and what it is protecting may not pose much of a problem for scientists or when there is only one or two CLs to use, the absence of transparent, comprehensive, and standardized CL names can become more of an issue with the increased use of CLs by resource managers and policy makers who have different priorities and goals, and with an increasing number of CLs within in a given park, forest, or airshed.

The main purpose of this study, therefore, is to review the elements that define and describe a CL and develop a standardized CL naming convention based on these elements. The goal is to produce a succinct, standardized naming system that can be applied to all types of CLs and clearly describes the CL, what it protects, and what distinguishes it from other types of CLs. The second purpose of this study is to further explore the elements that describe CLs to develop CL classes or a classification system. Such a classification system would group CLs (with standardized names) by common attributes to meet specific management objectives, such as species or ecosystem protection, and/or aid in setting CL values to protect specific terrestrial and/or aquatic biota. Lastly, this study will test the proposed CL naming convention and classification system by applying both to an existing US CL database and a US national forest case study location. Although important, this study does not evaluate CL exceedances with varying levels of deposition or potential impacts of historic, current, or future trends in deposition. Studies like Clark et al. (2018) provide excellent summaries of such trends in the United States.

## ELEMENTS OF A CL

The elements that describe a CL include attributes that: (1) *define* a CL, (2) describe the *method or model* used to estimate the CL, (3) indicate *where the CL is applicable*, and (4) acknowledge the study that is the *source of the CL*.

**FIGURE 1:**
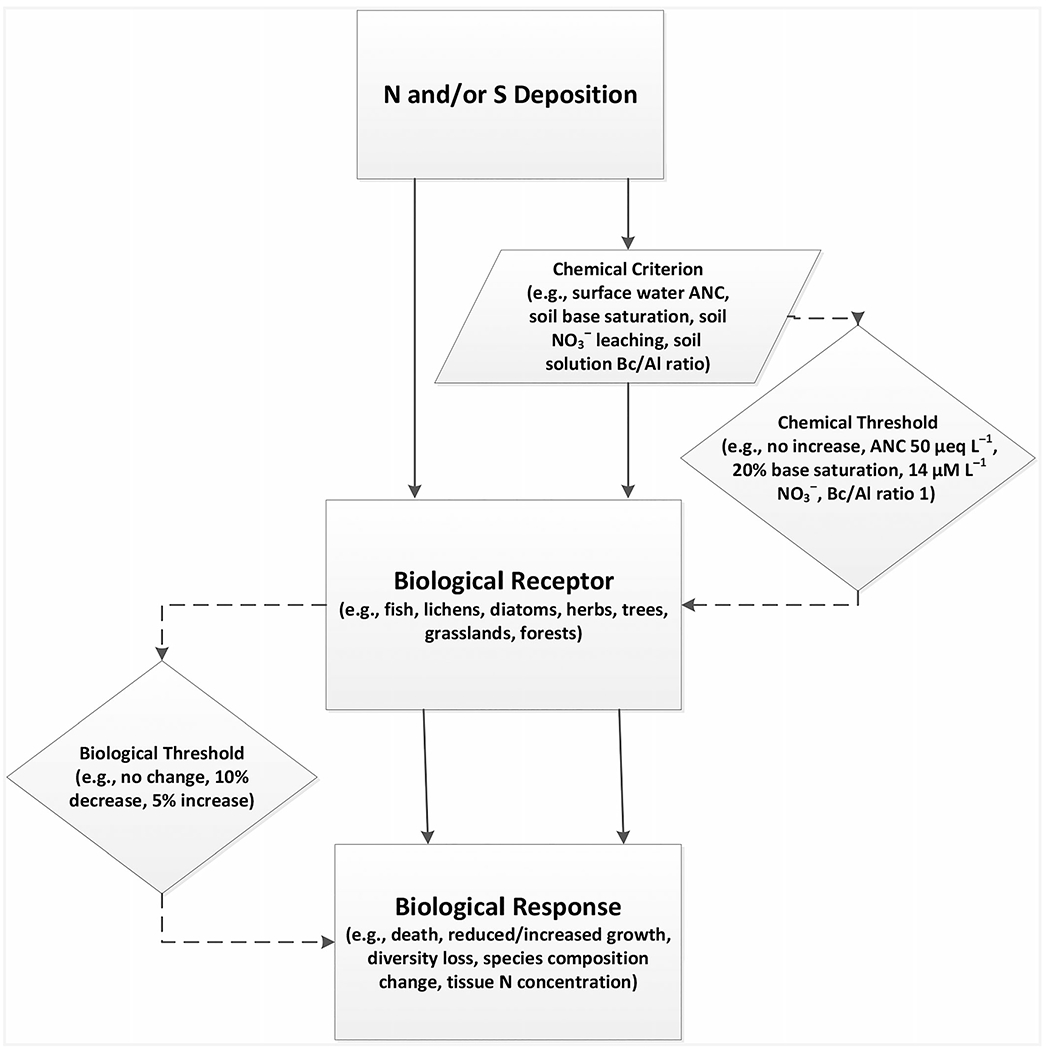
The components of a critical load (CL); modification of NADP–CLAD (2017). ANC, acid-neutralizing capacity; Bc/Al, base cations/aluminum; N, nitrogen; NO_3_^−^, nitrate; S, sulfur.

The components that (1) *define* a CL are outlined in Figure 1 (NADP–CLAD, 2017). Nitrogen and/or S deposition are the pollutants that act as the acidifying and/or eutrophying agents and determine the form of deposition for the CL. Therefore, it is possible to have a CL of N, CL of S, or CL of N + S.

The chemical criterion is the parameter of soil or surface water chemistry affected by deposition and experienced by the biological receptor in the ecosystem. It is the cause of the biological receptor changes in response to deposition. Common soil chemical criteria for terrestrial CLs of acidifying deposition include the ratio of nutrient base cations (calcium [Ca^2+^], potassium [K^+^], and magnesium [Mg^2+^]) to aluminum (Al) in the soil solution (i.e., Bc/Al), soil solution Ca/Al ratio, or soil percent base saturation (CLRTAP, 2017). Acid-neutralizing capacity (ANC), which represents the acid–base balance of a water body, is typically used to estimate surface water acidification CLs (CLRTAP, 2017), but other indicators such as pH are also used. Soil NO_3_^−^ leaching, or other measures of N cycling, are frequently associated with eutrophication or CLs of N (CLRTAP, 2017; Pardo et al., 2011). Although some empirical CLs are based on a relationship between deposition and soil NO_3_^−^ leaching, and therefore include the chemical criterion, many only relate deposition and biological response, so the chemical criterion is not measured and is often unknown and not explicitly identified.

The chemical threshold represents the value or level of the chemical criterion at which the biological receptor is considered to be at increased risk of being adversely impacted. This value is usually based on studies that evaluate the relationship between soil or water chemistry and the condition of biota. For example, a soil solution Bc/Al of 1.0 or 10.0 and 20% soil base saturation have been associated with reductions in tree growth or forest sustainability and are often used for forest acidification CLs (Arp et al., 1996; CLRTAP, 2017; NEG/ECP, 2001; Ouimet et al., 2006; Sverdrup & Warfvinge, 1993). Similarly, ANCs of 50 or 20 μeq L^−1^ are commonly used for surface water acidification CLs and are based on harm to aquatic biota (e.g., limited suitability of habitat for fish survival and reproduction) (Driscoll et al., 2001) or reduced health of brook trout associated with elevated concentrations of inorganic monomeric Al (Baldigo & Lawrence, 2007). For empirical CLs, where the chemical criterion and threshold are identified, increases or no change in N levels or process rates such as NO_3_^−^ leaching are commonly described as the CL threshold value (e.g., CLRTAP, 2017; Pardo et al., 2011). However, for these CLs, the relationship between chemical threshold and condition of biological receptor(s) in the system is usually unknown or not well understood. For example, an empirical CL of N deposition developed for a coniferous forest may be based on elevated levels of NO_3_^−^ leaching from the soil, which is indicative of excess N and eutrophication. Yet, there may not be any research or data linking the impaired condition of forest biota (e.g., reduced herbaceous species diversity or reduced growth of a tree species) with the increased NO_3_^−^ losses.

The biological receptor is the sensitive, or otherwise valued, element of the environment that is responsive to the conditions created by deposition and will be protected by the CL. The CL is developed for and protects the biological receptor against negative effects. The receptor can be an individual species (e.g., western hemlock [*Tsuga heterophylla*]), community of species and/or taxa (e.g., tree community), or a forest vegetation community consisting of trees and understory herbaceous plants.

The biological response and threshold are the measures of response (type and unit) used to characterize the harmful biological/ecological effects of deposition on the biological receptor. The type of response is commonly a metric associated with individual or community health such as growth, survival, tissue chemistry, community composition, detection frequency, abundance, or diversity, and the unit of measure is typically an “acceptable” amount of reduction (e.g., no change or 10% reduction) relative to a healthy or baseline condition. Species and/or communities differ in their sensitivities to the conditions created by deposition (Cronan & Grigal, 1995; Sverdrup & Warfvinge, 1993), and the forms and magnitudes of responses to different levels of N and/or S deposition can vary considerably among species or communities (Horn et al., 2018). For example, a CL of N for epiphytic macrolichens in forests, based on a 20% reduction in community richness, is 3.5 kg N ha^−1^ year^−1^ (Geiser et al., 2019), whereas the CL of N for a reduction in herbaceous species richness in open canopy systems ranges from 7.4 to 10.3 kg N ha^−1^ year^−1^ (Simkin et al., 2016).

In addition to the elements portrayed in Figure 1, a CL can be described by the (2) *method or model* used to estimate it. Two examples of commonly used methods/models are empirical and mass balance. As detailed earlier, empirical CLs are developed from dose–response or gradient studies that directly relate N and/or S deposition or additions to the response of a biological receptor or chemical indicator, while mass balance CLs are estimated using simple or complex models that use the difference of system inputs and outputs to determine the ability of an ecosystem to buffer the acidic impacts of deposition and protect sensitive biota from adverse changes in soil solution or surface water ANC or pH (CLRTAP, 2017). As empirical CLs are based on direct relationships between deposition and biological receptor or chemical criterion responses, they may be missing information about the mechanism or driver of the response or the specific sensitive biological receptor that is harmed by the changes in soil or water chemistry. In contrast, mass balance CLs are typically based on soil or water chemical thresholds with a connection to known chemistry–biological receptor response relationships.

Furthermore, a CL is represented by a spatial distribution or location (e.g., ecosystem, biome, or habitat type) of the biological receptor that informs (3) *where the CL is applicable*. For example, in Europe, CLs for different biota and receptors are associated with or assigned to EUNIS habitat classes (https://www.eea.europa.eu/data-and-maps/data/eunis-habitat-classification#tab-based-on-data). In the United States, Pardo et al. (2011) aggregated studies and assigned CLs of N by ecosystem type within Level I Ecoregions (Omernik, 1987). McNulty et al. (2007) estimated CLs of acidifying N and S deposition for 1-km forested areas, adjusting the CL for the forest type cover, coniferous versus broadleaf/deciduous. Similarly, Simkin et al. (2016) used individual site measurements to estimate CLs of N for herbaceous species diversity for open and closed canopies ecosystems that represent grasslands/shrublands and forests, respectively. Aquatic CLs are based on specific sampling location(s) where data were collected, but are assigned to represent the watershed, or in some cases, the headwater stream/lake or the lower stream network of the watershed that contains the sampling point(s). Examples of terrestrial and aquatic biogeographical classification systems in the United States that could be used to assign and represent single or aggregate similar CLs include biome (Kendeigh, 1961; Olson & Dinerstein, 1998), ecoregion (Bailey, 2016), vegetation (USNVC, 2017), land cover (USGS GAP, 2011; USGS NLCD, 2011; Yang et al., 2018), and watershed classifications (NHDPlus [National Hydrography Dataset Plus]; https://www.epa.gov/waterdata/nhdplus-national-hydrography-dataset-plus). A CL may be expanded and extrapolated across a defined geographical area (e.g., ecoregion or biome) if the biological receptor and/or ecosystem for which the CL is developed is present in the area.

Lastly, the study or *source of the CL* is also a descriptor of a CL. Research studies have been estimating CLs since the early 1990s, and some CLs for specific end points or ecosystems have since been updated with new models and data. For example, Pardo et al. (2011) developed CL of N estimates for herbaceous plants and shrubs for US Level I Ecoregions based on individual site studies. More recently, Simkin et al. (2016), Clark et al. (2019), and Wilkins et al. (2022) have produced more detailed CL of N estimates and response thresholds for herbaceous species based on nation-wide analyses of monitoring and measurement data across different ecosystems. Therefore, knowing the source and publication date of the CL may provide information regarding its scope, accuracy, and reliability. Information including the study citation and/or year could be used to describe the source of the CL.

In summary, the components that *define a CL*, the *method or model* used to develop it, the location(s) *where the CL is applicable*, and the study that is the *source of the CL* are the elements that could be used to develop a standardized CL naming convention and/or CL classification system. These elements describe what a CL represents, what it protects, and what distinguishes it from other CLs. In addition, these elements provide information that both scientists and resource managers may want to consider when applying a CL to achieve research or management goals. For the purposes of considering these elements side by side, Table 1 presents each element and its sub-elements, along with examples of descriptors from the CL literature that could be used to represent each element/sub-element in a standardized CL name or CL class.

## PROPOSED CL NAMING CONVENTION AND CL CLASSIFICATION SYSTEM

### Naming convention

Drawing from the elements outlined in Table 1, an ideal naming convention for CLs should balance the competing interests of information and length; it should contain enough detail to clearly communicate what it represents, yet be as succinct as possible to remain functional. In addition, it should prioritize elements that provide the essential information that defines a CL and clearly distinguishes it from other CLs, but not be overly specific or restrictive, so that it only applies to a single CL value. The CL name should contain information regarding the stressor or form of deposition (e.g., N or S) that is causing the negative impact. It should also indicate the sensitive biological receptor (e.g., herbaceous understory plants) that is harmed and protected by the CL. A naming system that includes these two elements could be applied to all the aforementioned herbaceous understory plant CLs developed by Pardo et al. (2011) and Simkin et al. (2016). In addition, a CL name could include information regarding the type and magnitude of biological response associated with the CL. A CL based on a 20% reduction in species richness may be more important to a resource manager than one based on an increase in tissue N concentrations. Similarly, it could be advantageous to know the soil or water chemistry criterion and threshold associated with the CL values. These elements not only provide information on how the CL was determined, but also provide an indication of the site conditions that could be used to determine which other site biota may be adversely impacted by the conditions created by deposition.

Conversely, there are other elements discussed above that may not be beneficial to include in a CL name. It may not be helpful to include the source (i.e., citation and year) of the CL in the standardized name because that would be too specific and result in each study having a unique CL name. Similarly, including location in the CL name may be too restrictive and result in more CL names than are useful. Instead, location could be used as a filter or a class to group CLs for a region or by location. CLs can be mapped and available as geospatial data layers that indicate their location and spatial extent, thereby allowing most users (e.g., natural resource managers or policy makers) to identify and summarize what CLs are present in their location of interest or geographical management unit. So, including the location in the CL name would be redundant.

Essentially, a CL name should clear about what is being affected by a specified form of deposition, how it is being harmed and, if known, the chemical conditions that are the cause or the driver of the change. Given these objectives, we propose the following components, in the specified order, for a standardized CL naming convention: (1) deposition form, (2) biological receptor (taxon and organizational level), (3) biological response + biological threshold, and/or (4) chemical criterion + chemical threshold (Example 1).

**Example 1.** deposition form – biological receptor – (biological response + biological threshold) ± (chemical criterion + chemical threshold).

Deposition form describes the pollutant(s) associated with the CL and the form(s) of deposition that are used in the calculation of CL exceedance. In addition, it allows for the easy grouping of CLs for pollutant sensitivity or risk analyses. Examples of deposition form within a CL name would be:

*CL of N or CL of N*+*S*

Biological receptor, represented by taxon and organizational level, clearly indicates what is being protected by the CL. Such information enables a resource manager or policy maker to formulate a decision regarding which CL(s) to use for the protection of a system. Furthermore, with a clear understanding of what each CL protects, resource managers can more easily communicate to stakeholders and other audiences what is being protected and what is being threatened by air pollution in a designated area. In addition, including biological receptor in the name highlights, by omission, what CLs are missing and where more information or research is needed. Within a CL name, the biological receptor would be denoted as an individual species common or scientific name (e.g., white pine) or a taxon category and organizational level (e.g., herbaceous community or fish species). Building onto the CL name, as an example, biological receptor would be presented as:

CL of N—Western hemlock

or

*CL of N* + *S—Fish community*.

Biological response + threshold clearly indicates how the biological receptor is negatively affected by deposition. Depending on management priorities, it may be important to know if the response is reduced growth versus reduced survival or increased soil NO_3_^−^ leaching versus change in community composition. Similarly, a CL may be based on a threshold of no reduction or a 20% reduction in species richness to protect the same biological receptor. However, the two options represent very different levels of protection (and potentially very different levels of deposition), and natural resource managers may want to know this information before selecting the CL(s) to meet their management goals. Examples of biological response and threshold added to the CL name could be:

CL of N—Western hemlock—20% reduction in growth

or

CL of N—Fish community—Reduced diversity.

Chemical criterion + threshold describes the mechanism causing the change or response in the biological receptor. The chemical criterion and threshold values used in CL calculations are based on established relationships with specific biological receptors and associated responses. As described earlier, a soil solution Bc/Al of 1.0 or 10.0 and 20% soil base saturation have been associated with reductions in tree growth or forest sustainability and are often used for forest acidification CLs (CLRTAP, 2017; Ouimet et al., 2006; Sverdrup & Warfvinge, 1993). Similarly, an ANC of 50 μeq L^−1^ is based, in part, on the relationship between declining fish species richness and ANC concentration across lakes in the Adirondack Mountains (Sullivan et al., 2006). However, CLs estimated using, for example, an ANC of 50 or Bc/Al of 10.0, not only protect the sites and species associated with the original relationships, but also offer protection to all biota that are adversely impacted by the soil or water chemistry threshold conditions. Therefore, by including the surface water chemical criterion and threshold in the CL name, it allows for the identification and protection of both the species included in the original chemistry–biological response relationships and other species and taxonomic groups that are sensitive to the soil and water chemistry conditions created by deposition. Examples of chemical criterion + threshold in the CL name could therefore include:

*CL of N + S—Fish community—Reduced diversity at 50 μeq L^−1^ ANC* (for a specific biological receptor)

or

*CL of N + S—Aquatic community—Reduced health at 50 μeq L^−1^ ANC* (for broader protection of aquatic community sensitive to water chemistry conditions).

The proposed standardized CL naming convention consisting of deposition form—biological receptor, response, and threshold—chemical criteria and threshold can be applied to all types of CLs. Some CLs may have all the information necessary and include all elements in their name, while others may not when certain information is unknown or not included in the CL determination. For example, a CL to protect coniferous forests from N + S deposition, and determined using a mass balance model and Bc/Al soil solution chemical criteria, includes all the elements and would be assigned a standardized name of *CL of N + S—Coniferous tree community—Reduced health at soil solution Bc/Al 1.0*. However, an empirical CL determined through the direct relationship between N deposition and reduced growth of Douglas fir (*Pseudotsuga menziesii*) trees does not include changes in the environmental driver (i.e., chemical criterion) because it is not known. So, the name for this CL would be, for example, *CL of N—Douglas fir—5% reduction in growth*. All individual CLs that contain the same specified elements/sub-elements and descriptors would be assigned the same CL name regardless of location or study source. Therefore, there may be multiple CL values from several studies and different locations with the same CL name.

### Classification system

As geospatial data have become the norm in natural resource management, most CLs have geographical references or locations and/or are mapped. Therefore, it is possible to identify and list the CLs that are contained within an area of interest or geographical management unit. Although assigning each CL a standardized name that clearly defines what the CL is, what it protects, and how it was determined may improve the situation by enabling CL users to make more informed decisions in the selection of CLs to meet management and protection goals, CL users may still be challenged by situations where their area of interest or geographical management unit contains many CLs to select from. In such situations, it may be helpful to adopt a classification system that groups the CLs by common attributes.

Historically, different CLs have been grouped by mechanism (e.g., CL of acidity, CL of eutrophication, or empirical CL). However, given the diversity of management objectives and resources in different geographical management units, it may be more advantageous to have a classification system that is not limited to a single criterion such as mechanism. Instead, the classification system could be more comprehensive, drawing from elements that define and describe the CL method, location, and/or source, and be highly flexible, consisting of one, a few, or multiple elements based on the needs of the user. Classes of CLs would not be “fixed” or predetermined. Instead, a class would be created by the user by choosing the elements to incorporate. Based on the geographical area of interest and specific management goals, the user would select which elements to include in their class of CLs. For example, a park manager may want to select all CLs that are available to protect the forest biome within their park. This class of CLs would produce a list of all the CLs for trees, lichens, shrubs, and herbaceous plants that are found within the park’s forests. From this list of CLs, the manager could then make an informed decision regarding which CL to use; they could select a CL to protect for a specific species or community, or they could adopt a protective lowest CL or 10th percentile CL that would be determined using all the CLs in the class. Similarly, a manager could explore CL class options by first building a broad class of CLs (e.g., all CLs in terrestrial systems) for a specific national park, and then further divide the CL class into separate classes by biome, biological receptor, or a combination of these elements, potentially including some of the elements that are also part of the standardized CL names.

Some geographical management units may only contain a limited number of CLs within their boundaries, and a CL classification may not be helpful in such situations. But, as more CLs are added to databases and/or if the area of interest is a large park, forest, or management region, there may be dozens of CLs to choose from, and a CL classification system may be a helpful tool to determine the CL(s) to apply for the protection of the geographical area of interest. Grouping CLs by classes can also help identify system, taxa, or biota of management interest that are missing or only have a few CLs, thereby identifying the need to acquire more information and potentially prioritize additional research.

## APPLICATION OF CL NAMING CONVENTION AND CLASSIFICATION SYSTEM TO EXISTING CL DATABASE AND CASE STUDY LOCATION

As a proof of concept and test of utility, we applied the proposed CL naming convention and classification system to an existing US CL database and to a US national forest case study location. The CL naming convention was applied to the CL database to evaluate the ability to assign standardized names to all the CLs in the database. In addition, we reviewed the database tables and fields to determine whether they: (1) had the core CL elements and sub-element (Table 1), and (2) contained other relevant elements or sub-elements that could be added as options for the CL classification system. The national forest case study was then carried out to determine if the CL names adequately described and distinguished the CLs for the national forest, and whether the proposed CL classification system could be used to group CLs into classes of potential use to the national forest managers. For the purposes of this study, the National Critical Load Database (NCLD) hosted by the National Atmospheric Deposition Program (NADP) under the auspices of the Critical Loads of Atmospheric Deposition (CLAD) science advisory committee (https://nadp.slh.wisc.edu/committees/clad/db/) served as the source of the CL database, and the Bridger-Teton National Forest in Wyoming, USA was the location of the case study.

### National Critical Load Database

The NADP–CLAD NCLD is the main CL database in the United States. It was started in the 2010s and consists of CLs produced by federal agencies, universities, and consultants, compiled in a consistent format for the continental United States (Lynch et al., 2022). Version 3.2 of the NCLD includes 2,757,206 geographically referenced CLs (see Appendix S1 for additional information) identified as forest soil CLs of acidity, surface water CLs of acidity, or empirical CLs for N or S (Lynch et al., 2022). Each of the three CL groups has its own set of descriptive and data tables with internally consistent fields and attributes. New CLs are routinely added to the NCLD, with each large addition resulting in a new version of the database.

The proposed CL naming convention and classification system was applied to the NADP–CLAD NCLD (version 3.2) by first reviewing the information contained within the NCLD tables and then summarizing the relevant CL naming convention and classification system elements, sub-elements, and descriptors (for each element/sub-element) into a series of tables using the structure outlined in Table 1 (Appendix S1: Table S1a–c). Most of the information for the CL naming convention and classification system was present in the NADP–CLAD NCLD. Although information from different tables and fields had to be combined, and source studies had to be reviewed to fill in missing data, it was possible to identify all the naming and classification elements and sub-elements for the CLs in the NCLD (Table 2). In addition, the NCLD contains a larger number of location-based sub-elements, thereby expanding the options for representing location within a CL classification system. However, as the NADP–CLAD NCLD consists of a variety of CLs developed over the course of 20 years using different methods and technologies, several difficulties in the application of the CL naming convention were encountered.

The NCLD contains CLs of acidifying N, S, and N + S deposition for forest and aquatic ecosystems. These types of CLs are typically calculated using simple mass balance models and soil and water chemistry data (i.e., chemical criterion + threshold), with varying degrees of connection to the original studies that established relationships between chemistry and biological receptors.

Sverdrup and Warfvinge (1993) conducted a meta-analysis of predominantly laboratory and seedling data and established the Bc/Al ratio at which root growth was reduced by 20%; a total of 30 coniferous and 22 broad-leaf species found in Europe and/or North America were evaluated. From this analysis, Sverdrup and Warfvinge (1993) concluded that a Bc/Al of ≥1.0 was suitable to protect European forests. NEG/ECP (2001) and Ouimet et al. (2006) evaluated the Bc/Al 1.0 ratio in relation to soil pH and base saturation and concluded that a Bc/Al ratio of 10.0 would maintain soil base saturation and provide better protection of the sustainability of forests in eastern Canada. Since these important studies, Bc/Al ratios of 1.0 and 10.0 have been broadly applied to conifer, broad-leaf, and/or mixed forests across the United States and Canada to calculate CLs of forest acidification, and three of these CL datasets are included in the NADP–CLAD NCLD (i.e., Duarte et al., 2013; McNulty et al., 2007; Phelan et al., 2014). Although the CL names that acknowledge the original sources of the chemical criterion and threshold (i.e., NEG/ECP, 2001; Ouimet et al., 2006; Sverdrup & Warfvinge, 1993) would be:

*CL of N + S—Tree species (one of the 55 species evaluated by* Sverdrup and Warfvinge (1993))—20% reduction in root growth at soil solution Bc/Al 1.0

and

CL of N + S—Tree community—Decreased sustainability with change in base saturation at soil solution Bc/Al 10.0,

the CLs in the three studies included in the NLCD were modeled to represent a broader level of protection to all tree species sensitive to the specified soil solution Bc/Al ratios. Therefore, these CLs in the NCLD were named:

CL of N + S—Tree community—Reduced health at soil solution Bc/Al 1.0

and

CL of N + S—Tree community—Reduced health at soil solution Bc/Al 10.0.

Similar challenges were experienced with the naming of the aquatic CLs of N, S, and N + S deposition in the NADP–CLAD NCLD. These CLs were determined using simple mass balance approaches and ecological impacts connected to independent studies for the region and not necessarily specific to the CL site.

Table 3 describes the ecological response to different levels of ANC based on the findings from a variety of studies. For example, when ANC is below 0 μeq L^−1^, acute impacts on all life-forms are expected, with a near-complete loss of fish populations, including nonacid native species such as brook trout (*Salvelinus fontinalis*), northern pike (*Esox lucius*), and others (Baldigo et al., 2018; Bulger et al., 2000; Sullivan et al., 2006). Aquatic diversity is at its lowest level, and the mechanism behind these losses has been attributed to elevated inorganic monomeric aluminum concentrations (Baldigo & Murdoch, 1997; Baldigo et al., 2019). At an ANC above 0 μeq L^−1^ but below 20 μeq L^−1^, aquatic diversity is impacted, with many species such as Atlantic salmon (*Salmo salar*) smolts and blacknose shiner (*Notropis heterolepis*) (Baldigo et al., 2018; Bulger et al., 2000; Liebich et al., 2011; Newman & Dolloff, 1995) missing, but comparatively acid-tolerant species such as brook trout are present with poor to moderately healthy populations. At an ANC between 20 and 50 μeq L^−1^, the fitness and population size of only sensitive species are impacted; acid-tolerant species can have healthy populations. ANC conditions from 50 to 100 μeq L^−1^ are considered suitable for brook trout and most fish species because buffering capacity is sufficient to prevent the likelihood of lethal episodic acidification events (Baker & Christensen, 1991; Driscoll et al., 2001). At ANC conditions above 100 μeq L^−1^, all biota are generally not affected. Collectively, these studies have, therefore, provided “thresholds” for CL estimates in watersheds throughout the United States. For example, Dupont et al. (2005), Scheffe et al. (2014), and McDonnell et al. (2014) used mass balance models for aquatic ecosystems to determine the amount of atmospheric N + S deposition to prevent ANC levels from decreasing below 20 to 50 μeq L^−1^. These ANC values were set to provide the corresponding levels of biological protection, without specific consideration of the aquatic species present in the water body of the watersheds. Therefore, although a more accurate CL name based on the original studies may be:

CL of N + S—Blacknose shiner—Reduced reproductive ability at ANC 20 μeq L^−1^,

the CLs in the NLCD represent a broader level of protection to all aquatic biota sensitive to stream or lake ANC of 20 μeq L^−1^ and were named:

CL of N + S—Aquatic biota community—Reduced health at ANC 20 μeq L^−1^.

The NADP–CLAD NCLD version 3.2 contains empirical CLs from multiple sources. Some are based on the results of individual field studies by ecoregion (i.e., Pardo et al., 2011), after a well-established method developed in Europe (Bobbink et al., 2003), while others are based on analyses of lichen, tree, and herbaceous datasets from monitoring networks and studies (Geiser et al., 2019; Simkin et al., 2016; Thomas et al., 2010). One of the challenges experienced with assigning standardized names to the NCLD empirical CLs was situations where multiple biological and chemical responses were used to develop the CL for a given ecosystem. For example, a CL from Pardo et al. (2011) combines: decreased growth of northern white cedar (*Thuja occidentalis*); decreased survivorship of yellow birch (*Betula alleghaniensis*), scarlet (*Quercus coccinea*) and chestnut (*Quercus montana*) oak, quaking aspen (*Populus tremuloides*), and basswood (*Tilia americana*); and increases in soil NO_3_^−^ leaching into a minimum and maximum CL of N for the Ecoregion Level I in which all of these species are found or responses were measured. Summarizing at this level allows researchers to make a conservative CL estimate to extrapolate to similar systems where research has not yet been conducted. However, multiple different biological responses and one chemical criterion that is not connected to biological responses make assigning a standardized CL name problematic according to the convention. The CL could be represented by multiple CL names, one for each of the identified biological responses and the chemical criterion, but this could lead to overrepresentation of the CL, and the CL values may not accurately represent the actual CL value associated with each of the individual responses and chemical criterion. Alternatively, the CL could be named for the most “representative” biological response or chemical criterion, as noted in the MetricSciName, FunctionalG, SensitivityClass, RecepI, RecepII, and Response Fields in the NCLD (Appendix S1: Table S1b). We chose this approach, and for the example given, produced the following CL name:

CL of N—Forest community—Reduction in tree species growth and/or survival.

This approach was applied to all CLs in the NCLD that were based on multiple biological responses and chemical criteria.

A second challenge with the empirical CLs in the NCLD was the CLs that were based only on a chemical criterion with no stated or apparent connection to a biological response. For example, one CL from Pardo et al. (2011) is based on NO_3_^−^ leaching from the soil to surface waters from various forest and chaparral communities in the Mediterranean California ecoregion using data from a series of studies (Breiner et al., 2007; Fenn et al., 2008, 2010, 2011; Fenn, Baron, et al., 2003; Fenn, Haueber, et al., 2003; Fenn & Poth, 1999; Fenn, Poth, et al., 2003; Meixner & Fenn, 2004). In this situation, only ecosystem-level biological receptors (i.e., coniferous forest and chaparral systems where the studies were conducted) and chemical criterion + threshold (i.e., increase in NO_3_^−^ leaching) are identified, and there is no clear understanding of the response or amount of change in the individual sensitive biological receptors in the forest and/or chaparral systems. The chemical response is associated with N loss to surface waters and elevated amounts in the soils, which may indicate N saturation and may lead to negative ecological effects such as reduced growth or changes in community composition, but these relationships were not measured. Therefore, the two standardized names assigned to this CL could not include a biological response and threshold and were:

CL of N—Forest community—Increase in NO_3_^−^ leaching

and

CL of N—Herbaceous plants and shrub community—Increase in NO_3_^−^ leaching.

These CLs can inform a land manager that the risk of “ecosystem change” from N deposition is likely, but the specifics or types or amounts of harm caused by the increased leaching are unclear. Therefore, the use of CLs like these may not be prioritized due to an inability to associate each CL with a specific sensitive biological receptor or clear management priority. Such CLs may indicate areas for future research and identification of biota that are harmed by high soil NO_3_^−^ levels.

Conversely, some of the empirical CLs in the NCLD are based on relationships between deposition and biological responses, where the chemical criterion causing the response is assumed, not measured. For example, Simkin et al. (2016) evaluated how herbaceous species richness in open and closed canopy systems changed along N deposition gradients across the United States. This analysis used environmental variation (temperature, precipitation, and soil pH) in the models, but did not use soil pH as a direct chemical criterion when establishing the CLs. The specific driver is unclear, but the assumption is that N deposition changes soil N, nutrient availability, and/or acid–base chemistry to a condition that either directly or indirectly impacts the growth and/or competitive ability of herbaceous plants, resulting in reduced species richness. For empirical CLs like this, we only had the biological receptor (i.e., herbaceous plant community), the biological response (i.e., species richness), and the biological threshold (i.e., reduction). The name for this CL example was therefore:

CL of N—Herbaceous plant community—Reduction in species richness.

While consistent with the research, the lack of a chemical criterion can create problems during the application of a CL where the distribution encompasses a variety of soil types and ecosystems. Understanding the mechanism behind the change in biology (i.e., change in soil chemistry driving the response) allows managers to know if the conditions underlying the CL relationship are applicable to the ecosystems in their forest or park.

Lastly, several of the thresholds associated with the empirical CL biological responses were described as “change.” For example, some CLs of N deposition in the NCLD are associated with a “change in foliar chemistry” and “change in the structure of mycorrhizal fungi.” Such thresholds are difficult to interpret, as reductions in deposition may cause a change in one direction, while increases in deposition may cause a change in the opposite direction. Most CL thresholds are associated with a specific direction of change in the condition of the biological receptor (e.g., increase or decrease). Although it is beyond the scope of this current study to review the original studies and determine what change occurred with increased deposition, future revisions to the NADP–CLAD NCLD and development of new CLs should attempt to use directional thresholds, when possible.

Although a series of challenges were encountered with the application of the proposed CL naming convention, all the CLs in the NADP–CLAD NCLD version 3.2 were successfully named. A total of 42 unique CL names for the 2,757,206 CLs (and 8,240,913 corresponding CL values) were determined. Tables 4 and 5 list the CL names for the NCLD-labeled forest soil and aquatic CLs of acidifying N, S, and N + S deposition and empirical CLs of N and S, respectively. Maintaining the current structure of the NADP–CLAD NCLD, these CL names could easily be added as a field(s) in the current database tables.

Table 2 also presents all the element and sub-element options for a CL classification system that could be applied to the CLs in the NADP–CLAD NCLD. As described earlier, the proposed CL classification system is not fixed. It is user-defined, with the CL class(es) being based on the elements selected by the user and their objectives or management priorities for a given location. Given the subset of NCLD CLs that are located within a defined geographical management unit, the user could select a class based only on deposition (e.g., N deposition) to list all CLs related to N deposition, or their selection could also include mechanism, biome, and biological receptor (e.g., Acidification—N + S Deposition—Mixed forest—Tree species) if the user’s interest is focused on protecting biota in a particular ecosystem that is sensitive to N deposition. Either way, the selected class would result in the listing of many, a few, or potentially none of the 33 different CLs (Tables 4 and 5) and all the associated CL values for the defined area. In situations where no CLs are listed for a class, this tells the user there are no CLs available to protect the biological receptors within the specified set of conditions and/or location. Many different CL classes could be produced for the 33 CLs in the NADP–CLAD NCLD, and it is beyond the scope of this article to explore, summarize, and recommend all of the best options for CL classes within the NCLD. Instead, the different classification options are explored in the next section with the application of the CL naming convention and classification system to the Bridger-Teton National Forest case study location.

### The Bridger-Teton National Forest

**FIGURE 2:**
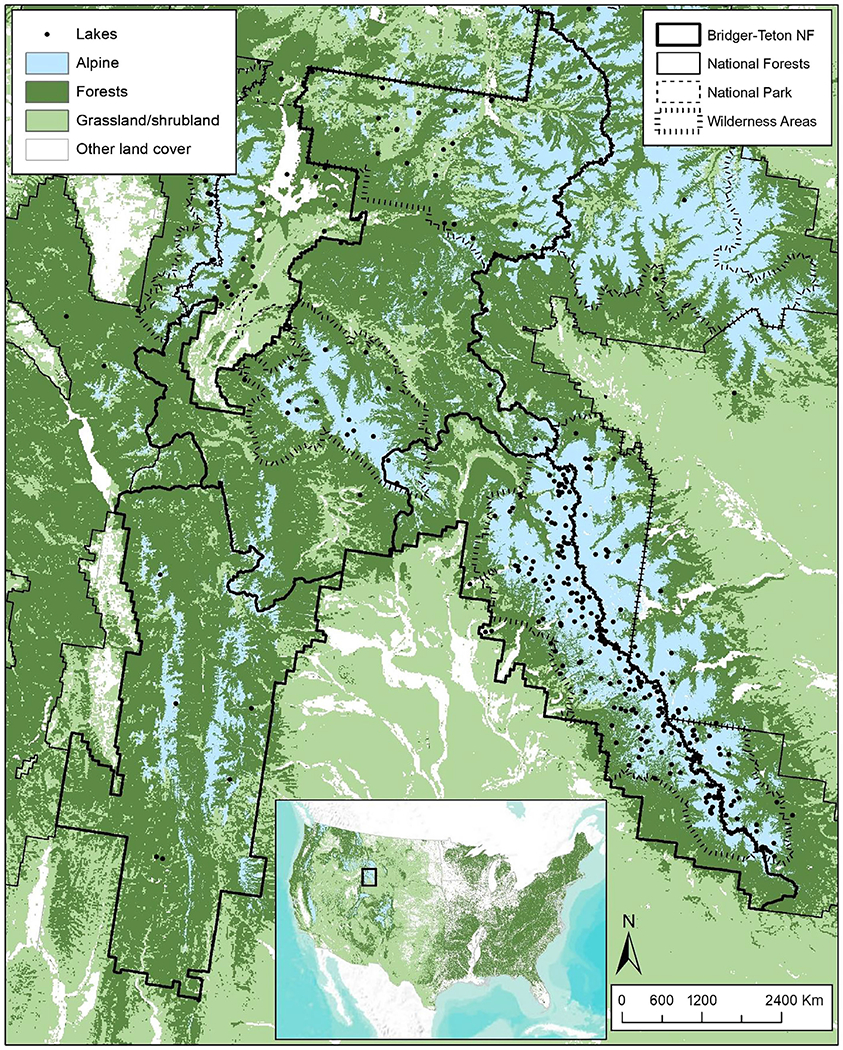
Location and ecosystems in Bridger-Teton National Forest (BTNF).

The 3.4 million-hectare Bridger-Teton National Forest (BTNF) is located in western Wyoming (Figure 2). The forest contains two Class I areas (Bridger Wilderness and Teton Wilderness) designated by the 1977 Clean Air Act amendments. This designation aids in the prevention of significant deterioration of air quality and air quality related values (AQRVs) due to major new or modified existing point sources of the six criteria pollutants (NO_x_, SO2, O_3_, Pb, PM_2.5_, and PM_10_). CLs are used by managers as deposition thresholds protective of AQRVs (sensitive air pollution receptors) and to assess risks to natural resources in forest planning and management (McMurray et al., 2021). In 2017–2019, the BTNF received estimated N and S deposition levels that ranged from 2.6 to 7.5 and 0.6 to 2.0 kg ha^−1^ year^−1^, respectively.

The BTNF contains a variety of terrestrial and aquatic ecosystems including alpine meadows and lakes, coniferous and broadleaf forests, rivers, and streams. It is home to a variety of plants, animals, and aquatic species sensitive to atmospheric N and S deposition. Based on the NADP–CLAD NCLD version 3.2, there are a total of 10,585 CL records and 31,718 individual CL values within the national forest boundaries, with the values ranging from 0 to 24,384 eq ha^−1^ year^−1^ for N, S, and N + S deposition. These CLs are from a total of six studies that estimated CLs at the scale of individual water bodies, 1-km grid cells, and Ecoregion Levels I and IV. With the application of the CL naming convention, these CLs are associated with a total of 24 standardized CL names (Table 6) that represent protection of aquatic, forest, tree, herbaceous plant and shrub, mycorrhizal fungal, and epiphytic lichen communities. The names also indicate to BTNF managers how each community could be harmed when deposition is above the CL values and alters surface water and soil chemistry. When the CLs are exceeded, the health of aquatic, forest, and tree communities decline; the composition of herbaceous plant, shrub, mycorrhizal fungal, and epiphytic lichen communities are altered; and species richness of herbaceous plant communities may be reduced. With this information, managers have a clear understanding of how different ecosystem components are impacted at different levels of N and S deposition.

Table 7 presents the elements that describe the CLs in the BTNF that could be used to classify or develop classes of CLs for the forest. The information for each element is from the NADP–CLAD NCLD version 3.2 (Lynch et al., 2022) and the individual studies that developed the CLs, if necessary. Elements describing the CL locations, including vegetation and ecosystem classes, were not included in Table 7 because these fields are yet to be populated within the NCLD. Name was not included because it is unique to each CL location (e.g., lake name) and not useful for classification purposes. Some of the CLs, for example, *CL of N + S—Tree community—Reduced health at soil solution Bc/Al 1.0*, did not have a biome or ecosystem type explicitly indicated in the NCLD. In these situations, the sources of the CL estimates (i.e., journal articles) were reviewed to determine the biome to assign to the CLs. Similarly, some of the CLs were developed at specific ecoregion levels. For example, *CL of N—Mycorrhizal fungal community—Change in structure* was estimated by Pardo et al. (2011) based on studies conducted within Level I Ecoregions, and *CL of N—Herbaceous plant community—Reduction in species richness* from Simkin et al. (2016) was summarized within the NCLD at Levels III or IV, with the ecoregion level being determined by a minimum number of study plots. For the purposes of CL classification, the locations of these CLs were only detailed at the ecoregion level for which they were derived to maintain consistency with their source and not overrepresent the CLs spatially.

**FIGURE 3:**
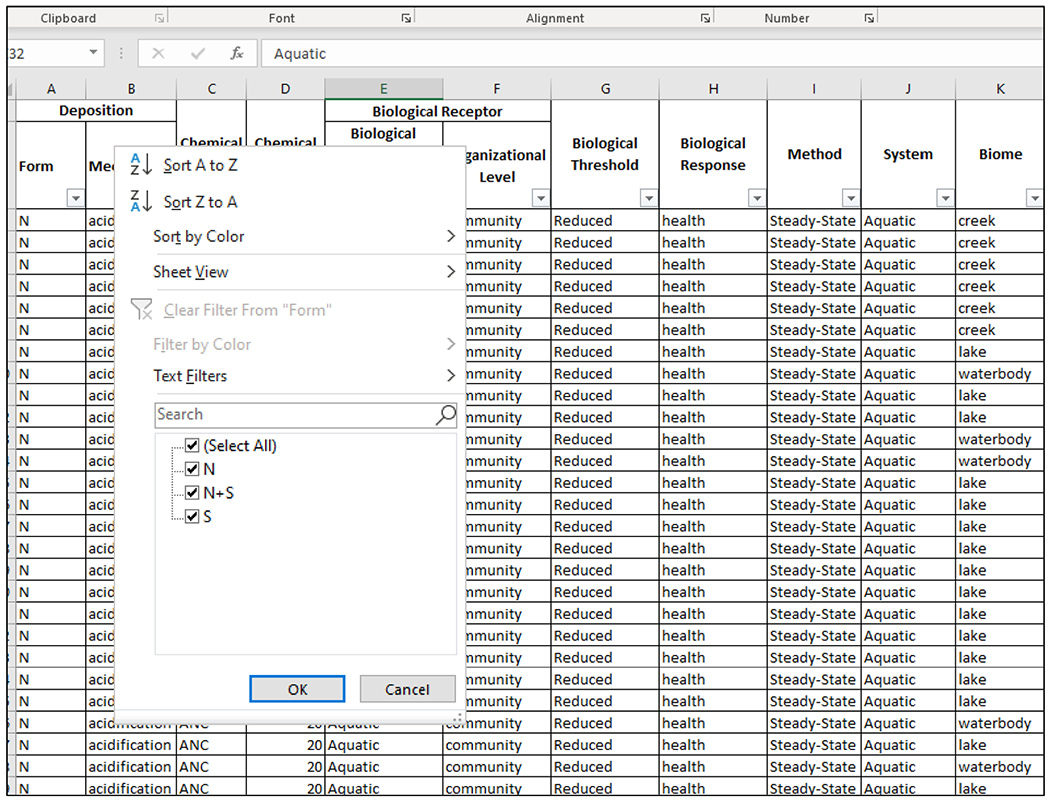
Example of critical load (CL) classes based on deposition form, using “filter” function on an Excel spreadsheet. N, nitrogen; S, sulfur.

**FIGURE 4:**
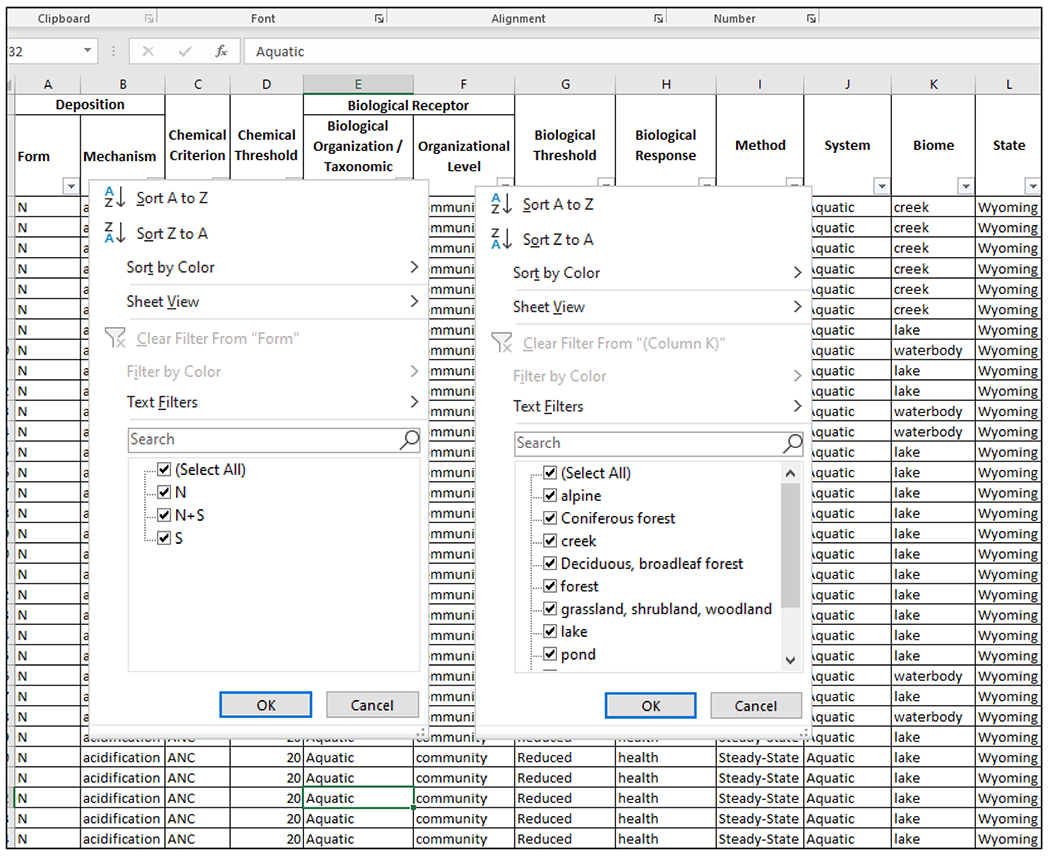
Example of critical load (CL) classes based on deposition form and biome, using “filter” function on an Excel spreadsheet. N, nitrogen; S, sulfur.

With the CL elements and associated information (Table 7) organized into a system, such as a simple excel spreadsheet, BTNF managers can explore and develop classes of CLs to support management and protection of the national forest and evaluate risks of current and future deposition to sensitive biota and ecosystems. For example, CLs could be classified by deposition form to evaluate the potential adverse impacts of elevated levels of N, S, or N + S deposition (Figure 3). Classes of CLs could also be developed by system type if, for example, a BTNF manager wanted to prioritize protection for aquatic or terrestrial systems. Similarly, CLs could be classified by deposition form and biome or ecoregion to determine the relative sensitivities of alpine, forest, and grassland systems within the forest boundaries to N, S, or N + S deposition (Figure 4). Using the CLs in the NADP–CLAD NCLD version 3.2 (Lynch et al., 2022) that are found in the BTNF, each CL class for the national forest could contain 1 to as many as 12 CLs and 31,689 CL values. For example, a CL class based on N deposition and forest biomes contains 7 CLs and 10,871 associated CL values. Although a class with only a single CL and value may not be useful, researchers are continually developing new CLs and updating the NADP–CLAD NCLD. There are currently plans to add individual tree and herb species, alpine aquatic lake, mycorrhizal fungi, and alpine vegetation CLs (Bowman et al., 2012; Clark et al., 2019; Horn et al., 2018; Lilleskov et al., 2019; Williams et al., 2017) to the future version(s) of the NCLD. Furthermore, CLs from studies unique to a forest, like BTNF (e.g., McMurray et al., 2021) or smaller geographical area (e.g., Symstad et al., 2019), can also be added to the CL naming and classification systems. Therefore, the number of CLs and CL values for BTNF and other areas managed for natural resources will continue to grow, further supporting the potential utility of the standardized CL naming convention and classification system.

## CONCLUSIONS

In this study, we successfully developed and tested a standardized CL naming convention that is applicable to CLs in a national database and a national forest in the United States. We also demonstrated how elements that describe a CL can be used to develop flexible, user-defined classes of CLs to support air quality risk assessment and prioritize natural resource management. Although challenges such as missing chemical criterion and/or biological indicator/response and mixing of multiple chemical criteria and biological indicators in single CLs were encountered, the results from this study demonstrate that adopting a naming convention that explicitly communicates what the CL is and what it protects is possible. It is therefore reasonable to conclude that the convention could also be applied to other CLs in the United States and other regions around the world. In addition, as the naming convention includes deposition, the soil or water chemistry that is impacted by deposition, and the biological receptor that is sensitive to the chemistry, it also provides guidance to scientists on what to consider and include when developing new CLs. Furthermore, the naming convention paired with grouping CLs into classes can also be used to identify data holes and prioritize future research to protect sensitive and valued natural resources.

## Supplementary Material

SOM

## Figures and Tables

**TABLE 1:** Critical load (CL) elements (e.g., deposition), sub-elements (e.g., mechanism), and examples of each (e.g., acidification) that could be used in a CL naming convention or classification system.

Element/sub-element	Example
Deposition	
Form	N; S; N + S
Mechanism	acidification; eutrophication; unknown

Chemical criterion	soil solution Bc/Al; soil percentage base saturation; surface water ANC; soil NO_3_^−^ leaching

Chemical threshold	10; 1; 20%; 0 μeq L^−1^; 20 μeq L^−1^; 40 μeq L^−1^; 50 μeq L^−1^; 100 μeq L^−1^; no increase

Biological receptor	
Biological organization/taxonomic group	forest; aquatic; tree; herbaceous plant; shrub; lichen; mycorrhiza; fungus; fish; diatom
Organizational level	species; community

Biological threshold	increase; decrease

Biological response	growth; survival; presence; abundance; % cover; species richness; diversity; tissue chemistry

Method/model	empirical; mass balance

Location	
System	terrestrial; aquatic
Biome	shrubland; grassland; forest; lake; pond; stream; river
Jurisdictional unit	state, county, district; national park, national forest, wildlife refuge, wilderness area; forest region, ecoregion

Source	
Year	year of publication
Citation	full citation

*Note*: The examples were derived from existing classification systems in the CL literature.

Abbreviations: ANC, acid-neutralizing capacity; Bc/Al, base cation/aluminum; N, nitrogen; NO_3_^−^, nitrate; S, sulfur.

**TABLE 2:** The critical load (CL) naming and classification system elements and sub-elements from the NADP–CLAD NCLD version 3.2 (Lynch et al., 2022).

Element/sub-element	Example
Deposition	
Form	N; S; N + S
Mechanism	acidification; eutrophication; unknown

Chemical criterion	soil solution Bc/Al; surface water ANC; soil NO_3_^−^ leaching; soil total N; soil net N mineralization; soil solution N; soil solution NO_3_^−^; N gas emissions; N cycling

Chemical threshold	10; 1; 20 μeq L^−1^; 40 μeq L^−1^; 50 μeq L^−1^; increase

Biological receptor	
Biological organization/taxonomic group	forest; aquatic; tree; shrub; herbaceous plants; herbaceous plants and shrubs; epiphytic lichen; mycorrhizal fungi
Organizational level	species; community

Biological threshold	reduction; increase; change

Biological response	growth; survival; composition; structure; activity; community/species richness; fine-root biomass; foliar chemistry; crown/canopy health; insect community; invasive species; thallus N concentration; health

Method	empirical; steady-state (mass balance)

Location	
System	terrestrial; aquatic
Biome	shrubland^a^; grassland^a^; woodland^a^; forest^a^; coniferous forest^a^; alpine^b^; serpentine grassland^b^; prairie^b^; tropical and subtropical forest^b^; lake^c^; pond^c^; stream creek^c^; river^c^; reservoir^c^; water body^c^
Ecoregion	EPA Ecoregion levels I–IV (Omernik, 1987)
State	50 states
Vegetation class	Classes I–III based on USNVC (1997) NLCD (Yang et al., 2018), USFS Forest Type (Ruefenacht et al., 2008) and/or USFS Landfire classification (USGS GAP, 2011)
Ecosystem class	USFS Ecoregion classes from Bailey (2016)
Name	Specific name of site or location including waterbody name (lake and stream), national forest or national park name (e.g., Adirondack Mountains, Boundary Waters Canoe Wilderness Area, etc.). For waterbodies, the name provided by the source of the data or NHDPlusV2.

*Note*: “Source” is not included in the table, as the NCLD contains 57 citations.

Abbreviations: ANC, acid-neutralizing capacity; Bc/Al, base cation/aluminum; CLAD, Critical Loads of Atmospheric Deposition; EPA, Environmental

Protection Agency; N, nitrogen; NADP, National Atmospheric Deposition Program; NCLD, National Critical Load Database; NO_3_^−^, nitrate; S, sulfur.

a From Vegetation Class I descriptions in tabs. 2C and 3C of NADP–CLAD NCLD version 3.2 (Lynch et al., 2022).

b From RecepII in tab. 2C of NADP–CLAD NCLD version 3.2 (Lynch et al., 2022).

c From Waterbody Type in tab. 2B of NADP–CLAD NCLD version 3.2 (Lynch et al., 2022).

**TABLE 3:** Aquatic status categories based on acid-neutralizing capacity (ANC) concentrations and corresponding health of aquatic biota (USEPA, 2009, 2011).

Category	Level	Expected biological effects
Acute concern	<0 μeq L^−1^	Complete loss of fish populations is expected. Planktonic communities have extremely low diversity and are dominated by acidophilic taxa. The number of individuals of plankton species that are present are greatly reduced.
Severe concern	0–20 μeq L^−1^	Highly sensitive to episodic acidification. During episodes of high acidifying deposition, brook trout populations may experience lethal effects. The diversity and distribution of zooplankton communities decline sharply.
Elevated concern	20–50 μeq L^−1^	Fish species richness is greatly reduced (i.e., more than half of expected species can be missing). On average, brook trout populations experience sublethal effects, including loss of health, ability to reproduce, and fitness. Diversity and distribution of zooplankton communities decline.
Moderate concern	50–100 μeq L^−1^	Fish species richness begins to decline (i.e., sensitive species are lost from lakes). Brook trout populations are sensitive and variable, with possible sublethal effects. Diversity and distribution of zooplankton communities also begin to decline as species that are sensitive to acidifying deposition are affected.
Low concern	>100 μeq L^−1^	Fish species richness may be unaffected. Reproducing brook trout populations are expected where habitat is suitable. Zooplankton communities are unaffected and exhibit expected diversity and distribution.

**TABLE 4:** Critical load (CL) naming convention applied to forest soil and surface water CLs of acidifying nitrogen (N), sulfur (S), and N + S deposition in the NADP–CLAD NCLD version 3.2 (Lynch et al., 2022).

NADP–CLAD NCLD critical load	Critical load name
Surface water critical loads for acidity	CL of S—Aquatic community—Reduced health at ANC 20 μeq L^−1^ CL of S—Aquatic community—Reduced health at ANC 40 μeq L^−1^ CL of S—Aquatic community—Reduced health at ANC 50 μeq L^−1^ CL of N—Aquatic community—Reduced health at ANC 20 μeq L^−1^ CL of N—Aquatic community—Reduced health at ANC 40 μeq L^−1^ CL of N—Aquatic community—Reduced health at ANC 50 μeq L^−1^ CL of N + S—Aquatic community—Reduced health at ANC 20 μeq L^−1^ CL of N + S—Aquatic community—Reduced health at ANC 40 μeq L^−1^ CL of N + S—Aquatic community—Reduced health at ANC 50 μeq L^−1^
Forest soil critical loads of acidity	CL of S—Tree community—Reduced health at soil solution Bc/Al 1.0 CL of N—Tree community—Reduced health at soil solution Bc/Al 1.0 CL of N + S—Tree community—Reduced health at soil solution Bc/Al 1.0 CL of S—Tree community—Reduced health at soil solution Bc/Al 10.0 CL of N—Tree community—Reduced health at soil solution Bc/Al 10.0 CL of N + S—Tree community—Reduced health at soil solution Bc/Al 10.0

Abbreviations: ANC, acid-neutralizing capacity; Bc/Al, base cation/aluminum; CLAD, Critical Loads of Atmospheric Deposition; NADP, National Atmospheric Deposition Program; NCLD, National Critical Load Database.

**TABLE 5:** Critical load (CL) naming convention applied to empirical CLs for nitrogen (N) and sulfur (S) in the NADP–CLAD NCLD version 3.2 (Lynch et al., 2022).

NADP–CLAD NCLD critical load	Critical load name
Empirical critical loads for nitrogen	CL of N—Forest community—Change in community composition CL of N—Forest community—Increase in soil N CL of N—Forest community—Increase in soil NO_3_^−^ leaching CL of N—Forest community—Reduction in tree species growth and/or survival CL of N—Tree community—Change in foliar chemistry CL of N—Tree community—Reduction in fine root biomass CL of N—Tree community—Reduction in species growth and/or survival CL of N—Herbaceous plants and shrub community—Increase in soil NO_3_^−^ leaching CL of N—Herbaceous plants and shrubs community—Change in composition CL of N—Herbaceous plants community—Change in composition CL of N—Herbaceous plants community—Increase in invasive species CL of N—Herbaceous plants community—Increase in soil NO_3_^−^ leaching CL of N—Herbaceous plants community—Reduction in species richness CL of N—Mycorrhizal fungal community—Change in composition CL of N—Mycorrhizal fungal community—Change in structure CL of N—Mycorrhizal fungal community—Reduction in activity CL of N—Mycorrhizal fungal community—Reduction in species richness CL of N—Epiphytic lichen community—Change in composition CL of N—Epiphytic lichen community—20% reduction in community richness CL of N—N-Sensitive epiphytic lichen community—20% reduction in community richness CL of N—Forage lichen community—20% reduction in species abundance CL of N—Cyanolichen community—20% reduction in species abundance
Empirical critical loads for sulfur	CL of S—Epiphytic lichen community—Change in composition CL of S—Epiphytic lichen community—20% reduction in community richness CL of S—S-Sensitive epiphytic lichen community—20% reduction in community richness CL of S—Forage lichen community—20% reduction in species abundance CL of S—Cyanolichen community—20% reduction in species abundance

Abbreviation: CLAD, Critical Loads of Atmospheric Deposition; NADP, National Atmospheric Deposition Program; NCLD, National Critical Load Database; NO_3_^−^, nitrate.

**TABLE 6:** Critical loads (CLs) of nitrogen (N), sulfur (S), and N + S deposition (from the NADP–CLAD NCLD version 3.2; Lynch et al., 2022) in the Bridger-Teton National Forest (BTNF).

Critical load name	No. critical load values	Critical load scale	Range of critical load values (eq ha^−2^ year^−1^)	Source of critical loads
CL of S—Aquatic community—Reduced health at ANC 20 μeq L^−1^	272	Water body	0–24,287	Lynch et al. (2020), Scheffe et al. (2014)
CL of N—Aquatic community—Reduced health at ANC 20 μeq L^−1^	272	Water body	984–24,394	Lynch et al. (2020), Scheffe et al. (2014)
CL of N + S—Aquatic community—Reduced health at ANC 20 μeq L^−1^	272	Water body	984–24,394	Lynch et al. (2020), Scheffe et al. (2014)
CL of S—Tree community—Reduced health at soil solution Bc/Al 1.0	9362	1-km grid	589–3786	McNulty et al. (2013)
CL of N—Tree community—Reduced health at soil solution Bc/Al 1.0	9362	1-km grid	736–3829	McNulty et al. (2013)
CL of N + S—Tree community—Reduced health at soil solution Bc/Al 1.0	9362	1-km grid	736–3829	McNulty et al. (2013)
CL of S—Tree community—Reduced health at soil solution Bc/Al 10.0	929	1-km grid	387–1970	McNulty et al. (2013)
CL of N—Tree community—Reduced health at soil solution Bc/Al 10.0	929	1-km grid	520–2103	McNulty et al. (2013)
CL of N + S—Tree community—Reduced health at soil solution Bc/Al 10.0	929	1-km grid	520–2103	McNulty et al. (2013)
CL of N—Forest community—Increase in soil N	2	Level I Ecoregion	286–1213	Pardo et al. (2011)
CL of N—Forest community—Increase in NO_3_^−^ leaching	2	Level I Ecoregion	286–1213	Pardo et al. (2011)
CL of N—Herbaceous plants and shrubs community—Change in composition	4	Level I Ecoregion	214–714	Pardo et al. (2011)
CL of N—Herbaceous plant community—Reduction in species richness	5	Level IV Ecoregion	552–981	Simkin et al. (2016)
CL of N—Mycorrhizal fungal community—Change in structure	2	Level I Ecoregion	357–714	Pardo et al. (2011)
CL of N—Epiphytic lichen community—Change in composition	5	Level I Ecoregion; 1-km grid	107–507	Pardo et al. (2011), Geiser et al. (2019)
CL of N—Epiphytic lichen community—20% reduction in community richness	1	1-km grid	250	Geiser et al. (2019)
CL of N—N-sensitive epiphytic lichen community—20% reduction in community richness	1	1-km grid	221	Geiser et al. (2019)
CL of N—Forage lichen community—20% reduction in species abundance	1	1-km grid	136	Geiser et al. (2019)
CL of N—Cyanolichen community—20% reduction in species abundance	1	1-km grid	93	Geiser et al. (2019)
CL of S—Epiphytic lichen community—Change in composition	1	1-km grid	168	Geiser et al. (2019)
CL of S—Epiphytic lichen community—20% reduction in community richness	1	1-km grid	374	Geiser et al. (2019)
CL of S—S-sensitive epiphytic lichen community—20% reduction in community richness	1	1-km grid	156	Geiser et al. (2019)
CL of S—Forage lichen community—20% reduction in species abundance	1	1-km grid	162	Geiser et al. (2019)
CL of S—Cyanolichen community—20% reduction in species abundance	1	1-km grid	143	Geiser et al. (2019)
Total	31,718		0–24,394	

Abbreviations: ANC, acid-neutralizing capacity; Bc/Al, base cation/aluminum; CLAD, Critical Loads of Atmospheric Deposition; NADP, National Atmospheric Deposition Program; NCLD, National Critical Load Database; NO_3_^−^, nitrate.

**TABLE 7:** The elements and sub-elements associated with Bridger-Teton National Forest critical loads (CLs) (from the NADP–CLAD NCLD version 3.2; Lynch et al., 2022).

Element/sub-element	Example
Deposition	
Form	N; S; N + S
Mechanism	acidification; eutrophication; unknown

Chemical criterion	soil solution Bc/Al; surface water ANC; soil NO_3_^−^ leaching; soil total N

Chemical threshold	10; 1; 20 μeq L^−1^; increase

Biological receptor	
Biological organization/taxonomic group	forest; aquatic; tree; herbaceous plants; herbaceous plants and shrubs; mycorrhizal fungi; epiphytic lichen; N-sensitive epiphytic lichen; S-sensitive epiphytic lichen; forage lichen; cyanolichen
Organizational level	community

Biological threshold	reduction; change; 20% reduction

Biological response	composition; structure; species richness; health; community richness; species abundance

Method	empirical; steady-state (mass balance)

Location	
System	terrestrial; aquatic
Biome	shrubland; grassland, shrubland, woodland; forest; coniferous forest; deciduous, broadleaf forest; alpine; lake; pond; creek; river; water body; closed canopy system (forest)
State	Wyoming
Jurisdiction	National forest
Ecoregion Level I	North American Deserts; Western Forested Mountains
Ecoregion Level II	Western Cordillera; Cold Deserts
Ecoregion Level III	Middle Rockies; Wyoming Basin
Ecoregion Level IV	Absaroka Volcanic Subalpine Zone; Absaroka-Gallatin Volcanic Mountains; Alpine Zone; Foothill Shrublands and Low Mountains; Granitic Subalpine Zone; High Elevation Valleys; Mid-elevation Sedimentary Mountains; Powder River Basin; Sedimentary Subalpine Zone; Semiarid Benchlands and Canyonlands; Yellowstone Plateau

Abbreviations: ANC, acid-neutralizing capacity; Bc/Al, base cation/aluminum; CLAD, Critical Loads of Atmospheric Deposition; N, nitrogen; NADP, National Atmospheric Deposition Program; NCLD, National Critical Load Database; NO_3_^−^, nitrate; S, sulfur.

## Data Availability

No new data were collected for this study.
