# Supplementary material for: Standardized naming convention and classification system for critical loads of nitrogen and sulfur deposition
Source: Ecosphere. Author manuscript; Available in PMC 2025 Jan 27. (PMC11770556; doi:10.1002/ecs2.4473)
Supplement: SOM [file NIHMS2036908-supplement-SOM.docx]

**Appendix S1**

**Title:** Standardized naming convention and classification system for critical loads of nitrogen and sulfur deposition

**Authors:**

Jennifer Phelan^1^, Michael D. Bell^2^, Jason A. Lynch^3^, and Linda H. Geiser^4^

^1^corresponding author – jenphelan@rti.org; RTI International, 3040 Cornwallis Rd., RTP, NC 27709-2194
^2^National Park Service – Air Resources Division, Lakewood, CO 80235
^3^U.S. Environmental Protection Agency – Office of Air and Radiation, Office of Atmospheric Protection, Washington, DC 20460
^4^U.S. Department of Agriculture – Forest Service, Air Resource Management, Washington, DC 20227

**Journal:** Ecosphere

For this current study, Tables 3(A-C) of the National Atmospheric Deposition Program – Critical Loads of Atmospheric Deposition Science Committee National Critical Load Database (NADP–CLAD NCLD) v3.2 were the source of the method fields (Lynch et al., 2022). The deposition fields were from NCLD Tables 1 and 2 (A-C), and Tables 1 (A-C), Tables 2 (B-C) and 3 C were the sources of the location fields. Tables 2 (A-B) and 3B were the sources of chemical criterion and threshold for the Forest Soil and Surface Water critical loads (CLs) of Acidity, and Table 2C was the source of the biological receptor, response, and threshold fields for the Empirical CLs for N and S. Table 2C was the source of the CL description. See Lynch et al. (2022) for definitions of abbreviations used in tables.

Table S1 a-c. Potential critical load (CL) naming and classification elements and sub-elements from the NADP–CLAD NCLD v3.2 (Lynch et al., 2022).

1. Method, Deposition, and Location elements.

1. Chemical criterion and threshold and biological receptor, response, and threshold elements.

^1^ Changed from “mortality” (in Table 2C of NADP–CLAD NCLD (Lynch et al., (2022)) to “survival” to correct representation of response.

1. Detailed critical load (CL) descriptions.

There are several additional things to note about the use of the NADP-CLAD NCLD v3.2 (Lynch et al., 2022) in this study. 1. The NADP–CLAD NCLD v3.2 also contains target loads. A target load is “the deposition load that is selected or determined to provide a level of protection for or recovery of sensitive ecosystem components based on time frame for resource protection, feasibility of emissions reductions, and/or other considerations” (NADP–CLAD, 2017). Target load elements are included in Tables S1(a-c), but are not included in the CL count and the subsequent development of standardized names and classification system for the NCLD that are outlined in the main text. It may be possible to add target loads to the convention and system, but additional elements including “target load” and “year” would need to be included in the naming convention to avoid confusion between the critical and target loads. 2. In some cases, the geographically-referenced CLs in the NADP-CLAD NCLD v3.2 consist of more than one CL value. Each geographically-referenced CL may consist of N and S CLs and/or multiple estimates of N and/or S CLs (e.g., CLminN, CLmaxN, and CLmaxS for forest CLs; CLminN, CLS, and CLNSce for water CLs; and CLmin and CLmax for empirical CLs of N and CLS for empirical CLs of S). In these situations, because the CL values are associated with the same sensitive receptor/set of receptors in the same physical location, the CLs are only represented as a single count in the 2,757,206 value reported in the main text. A total of 8,240,913 CL values correspond to the 2,757,206 geographically-referenced CLs. Similarly, in the application of the NADP–CLAD NCLD v3.2 to the Bridger Teton National Forest (BTNF), there are 10,585 CL records (each for a specific biological receptor/set of receptors in a specific geographical location) and 31,718 individual CL values in the boundaries of the national forest. Each record may have N and S CLs and/or multiple estimates of N and/or S CLs (e.g., CLminN, CLmaxN, and CLmaxS for forest CLs; CLminN, CLS, and CLNS for surface water CLs; and CLmin and CLmax for empirical CLs of N and CLS for empirical CLs of S). 3. A series of CLs for individual lichen species are included in the NADP–CLAD NCLD v3.2. However, these data have not been spatially defined yet. Therefore, although elements for these individual species CLs are included in Tables S1(a-c) that describe the elements from the NADP–CLAD NCLD v3.2, they are not included in the CL count and the subsequent development of standardized names and classification system for the NCLD outlined in the main text.

**References:**

Bailey, R. G. 2016. “Bailey's ecoregions and subregions of the United States, Puerto Rico, and the U.S. Virgin Islands.” *Fort Collins, CO: Forest Service Research Data Archive*. <https://doi.org/10.2737/RDS-2016-0003>.

Lynch, J.A., Phelan, J., Pardo, L.H., McDonnell, T.C., Clark, C.M., Bell, M.D., Geiser, L.H., and Smith, R.J. 2022. Detailed Documentation of the National Critical Load Database (NCLD) for U.S. Critical Loads of Sulfur and Nitrogen, version 3.2, National Atmospheric Deposition Program, Wisconsin State Laboratory of Hygiene, Madison, WI.

NADP–CLAD (National Atmospheric Deposition Program Critical Loads of Atmospheric Deposition Science Committee). 2017. CLAD Critical Load Definitions, version 1.1 2017. CL AD Critical Load Definitions, version 1.1. NADP Data Report 2016-01. Illinois State Water Survey, University of Illinois at Urbana-Champaign, IL.

Omernik, J.M. 1987. “Ecoregions of the conterminous United States. Map (scale 1:7,500,000).” *Annals of the Association of American Geographers* **77**: 118-25.

[Ruefenacht](https://pubag.nal.usda.gov/?q=%22B.+Ruefenacht%22&search_field=author), B., [Finco](https://pubag.nal.usda.gov/?q=%22M.V.+Finco%22&search_field=author), M. V., [Nelson](https://pubag.nal.usda.gov/?q=%22M.D.+Nelson%22&search_field=author), M. D., [Czaplewski](https://pubag.nal.usda.gov/?q=%22R.+Czaplewski%22&search_field=author), R., [Helmer](https://pubag.nal.usda.gov/?q=%22E.H.+Helmer%22&search_field=author), E. H., [Blackard](https://pubag.nal.usda.gov/?q=%22J.A.+Blackard%22&search_field=author), J. A., [Holden](https://pubag.nal.usda.gov/?q=%22G.R.+Holden%22&search_field=author), G.R., [Lister](https://pubag.nal.usda.gov/?q=%22A.J.+Lister%22&search_field=author), A.J., [Salajanu](https://pubag.nal.usda.gov/?q=%22D.+Salajanu%22&search_field=author), D., [Weyermann](https://pubag.nal.usda.gov/?q=%22D.+Weyermann%22&search_field=author), D., and [K. Winterberger](https://pubag.nal.usda.gov/?q=%22K.+Winterberger%22&search_field=author). 2008. “[Photogrammetric engineering and remote sensing.” **74**:](https://pubag.nal.usda.gov/?f%5Bjournal_name%5D%5B%5D=Photogrammetric+engineering+and+remote+sensing&f%5Bpublication_year_rev%5D%5B%5D=7992-2008&f%5Bsource%5D%5B%5D=2008+v.74+no.11) 1379-88.

USGS GAP (U.S. Geological Survey Gap Analysis Program). 2011. “U.S. Geological Survey Gap Analysis Program”, 20160513, GAP/LANDFIRE National Terrestrial Ecosystems 2011: U.S. Geological Survey, <https://doi.org/10.5066/F7ZS2TM0>.

USNVC (U.S. National Vegetation Classification). 2017. “United States National Vegetation Classification Database”, V2.01. Federal Geographic Data Committee, Vegetation Subcommittee, Washington DC.

Yang, L., Jin, S., Danielson, P., Homer, C., Gass, L., Bender, S., Case, A., Costello, C., Dewitz, J., Fry, J., Funk, M., Granneman, B., Liknes, G., Rigge, M., and G. Xian. 2018. “A new generation of the United States National Land Cover Database: Requirements, research priorities, design, and implementation strategies.” *ISPRS Journal of Photogrammetry and Remote Sensing* **146**: 108-23.
